# Ferulic Acid Metabolites Attenuate LPS-Induced Inflammatory Response in Enterocyte-like Cells

**DOI:** 10.3390/nu13093152

**Published:** 2021-09-10

**Authors:** Gabriele Serreli, Micaela Rita Naitza, Sonia Zodio, Vera Piera Leoni, Martina Spada, Maria Paola Melis, Anna Boronat, Monica Deiana

**Affiliations:** 1 Department of Biomedical Sciences, University of Cagliari, Cittadella Universitaria SS 554, 09042 Monserrato, Italy; gabriele.serreli@unica.it (G.S.); michinaitza@gmail.com (M.R.N.); sonia.zodio@libero.it (S.Z.); vleoni@unica.it (V.P.L.); martina.spada@unica.it (M.S.); mpmelis@unica.it (M.P.M.); 2 Integrative Pharmacology and Systems Neurosciences Research Group, Hospital del Mar Medical Research Institute, 08003 Barcelona, Spain; aboronat@imim.es

**Keywords:** inflammation, lipopolysaccharide, nitric oxide, nitric oxide synthase, intestinal cells, MAP kinases, metabolites, polyphenols

## Abstract

Ferulic acid (FA) is a polyphenol pertaining to the class of hydroxycinnamic acids present in numerous foods of a plant origin. Its dietary consumption leads to the formation of several phase I and II metabolites in vivo, which represent the largest amount of ferulates in the circulation and in the intestine in comparison with FA itself. In this work, we evaluated their efficacy against the proinflammatory effects induced by lipopolysaccharide (LPS) in intestinal Caco-2 cell monolayers, as well as the mechanisms underlying their protective action. LPS-induced overexpression of proinflammatory enzymes such as inducible nitric oxide synthase (iNOS) and the consequent hyperproduction of nitric oxide (NO) and cyclic guanosine monophosphate (cGMP) were limited by physiological relevant concentrations (1 µM) of FA, its derivatives isoferulic acid (IFA) and dihydroferulic acid (DHFA), and their glucuronidated and sulfated metabolites, which acted upstream by limiting the activation of MAPK p38 and ERK and of Akt kinase, thus decreasing the nuclear factor kappa-light-chain-enhancer of activated B cells (NF-ĸB) translocation into the nucleus. Furthermore, the compounds were found to promote the expression of Nrf2, which may have contributed to the downregulation of NF-ĸB activity. The overall data show that phase I/II metabolites retain the efficacy of their dietary free form in contrasting inflammatory response.

## 1. Introduction

The intestinal epithelium is a wide biological interface dedicated to the absorption of nutrients and water, of which homeostasis is regulated by several factors such local and systemic immune factors, and by its microbial content. The dysregulation in the gut of all of these factors is known to be partly responsible for the pathogenesis of inflammatory bowel diseases (IBD) and cancer [1]. Cytokines and Gram-negative bacterial lipopolysaccharide (LPS), in particular, can induce permeability dysfunctions through activation of the immune system and inflammatory processes [2]. Furthermore, intestinal inflammation induced by these noxious molecules is characterized by an aberrant activation of redox-sensitive signaling pathways such as the phosphatidylinositol 3 kinase/protein kinase B (PI3K/Akt) and the mitogen-activated protein kinases (MAPKs), which include the extracellular signal-regulated kinase (ERK), p38, and the c-Jun N-terminal kinase (JNK). All of these kinases can in turn lead to dysregulation of the inflammatory response involving the nuclear factor kappa-light-chain-enhancer of activated B cells (NF-ĸB) pathway [3,4]. All of these upstream processes are pivotal in the expression of typical inflammatory enzymes such as inducible nitric oxide synthase (iNOS) and cyclooxygenase-2 (COX-2) [5]. The former is mostly transcriptionally regulated and is not usually expressed in most cells [6]. Its expression is commonly induced by cytokines in almost every cell and it generates a locally high level of nitric oxide (NO) for prolonged periods of time [7]. In the intestinal epithelium, NO may be released at relatively low concentrations in order to exert protective effects against pathogens, whereas a chronic and excessive release leads to deleterious effects. Indeed, at high concentrations, NO combines rapidly with superoxide (O_2_^−^) anions to form peroxynitrite (ONOO^−^), and with this powerful oxidant it contributes to oxidative stress and inflammation maintenance [8]. Moreover, iNOS activation elicits the formation of cyclic guanosine monophosphate (cGMP) through the NO/cGMP pathway in the presence of soluble guanylate cyclase (sGC). The abnormal cGMP production, after iNOS overexpression at the intestinal level, is known to contribute to enhancing permeability and promoting diarrhea in ulcerative colitis [9]. Under pathological conditions, the production of NO and cGMP is often upregulated, due to an enhancement of iNOS expression. This condition generates a loss of barrier function with epithelial derangement and bacterial translocation, which give rise to inflammation [10]. It is also known that there is crosstalk between the transcription factor nuclear factor erythroid 2-related factor 2 (Nrf2) and the NF-ĸB signaling pathways to control the transcription and activity of downstream target proteins like the aforementioned iNOS [11]. The inactivation or suppression of the NF-ĸB-mediated transcriptional activity through Nrf2 likely takes place in the early stages of inflammation, resulting in reduced intestinal mucosal damage and tight junction (TJ) impairment [11].

As the most important site of absorption of exogenous substances, the intestine endures the effects not only of harmful substances, but also of positive bioactive compounds derived from the diet. Compounds such as the so-called polyphenols, present in large quantities in many foods of a plant origin, are extensively investigated for their anti-inflammatory and antioxidant properties [12], which can be exerted especially in the intestine, where they concentrate before absorption, following the ingestion of foods naturally containing them [13]. Among these substances, one of the most studied is ferulic acid (FA), which has been shown to possess several beneficial properties for maintaining cellular homeostasis [14]. FA is usually found in foods like rice, oats, wheat, vegetables, grasses, fruits, grains, seeds of coffee, flowers, and nuts [15]. The preventive and/or therapeutic efficacy of FA is dependent on its physiological concentrations, which are predominated by its pharmacokinetic properties and especially by its metabolism. Several metabolic studies have indeed shown that FA is metabolized in vivo into a number of metabolites, including FA sulfate (sulf), FA glucuronide (glu), FA sulfoglucuronide (FA diconjugate with sulfate and glucuronide), FA diglucuronide, feruloylglycine, dihydroferulic acid (DHFA), isoferulic acid (IFA), and IFA sulfate [16,17]. The conjugation of FA occurs mainly in the liver and in the intestinal mucosa by phase I/II metabolism, but further metabolization by the microbiota may also take place in the gut [18]. Once formed, these metabolites can reach physiologically relevant concentrations in the intestine and exert positive biological effects [18]. Despite this, these compounds have been poorly investigated with respect to their potential effect, which is normally attributed to their parent compounds.

The purposes of the present research fit into this context, with the main objective of studying the efficacy of these metabolites as molecules with and anti-inflammatory activity in an in vitro experimental model of the intestinal barrier. Here, differentiated Caco-2 cell monolayers were treated with pathological concentrations of LPS (1 µg/mL), which is able to stimulate the inflammatory response in intestinal cells, and were challenged with FA, together with its metabolites DHFA, IFA, FA glu, DHFA glu, and IFA sulf, at a concentration of 1 µM. We focused in particular on (a) the expression of iNOS and release of NO and cGMP, (b) the modulation of NF-ĸB involving Akt and MAPK pathways, and (c) the expression of the antioxidant transcription factor Nrf2.

## 2. Materials and Methods

### 2.1. Chemicals

Bradford reagent, dimethyl sulfoxide (DMSO), LPS from *Escherichia coli*, and Thiazolyl Blue Tetrazolium Bromide (MTT) were purchased from Sigma Aldrich (Milan, Italy). Cell culture materials were obtained from Invitrogen (Milan, Italy). FA, FA glu, DHFA, DHFA glu, IFA, and IFA sulf were obtained from LGC standards (Sesto San Giovanni, Italy). Each compound was dissolved in methanol (stock solution 0.1 mg/mL) and kept at −20 °C. The LPS solutions were prepared in 1 mg/mL, in water just before their use for treatments.

### 2.2. Cell Cultures

Intestinal Caco-2 cells (ECACC Salisbury, Wiltshire, UK) were cultured in Dulbecco’s modified Eagle’s medium (DMEM), supplemented with 10% heat-inactivated bovine serum, 100 U/mL penicillin, 100 mg/mL streptomycin, 1% non-essential amino acids, and 2 mM L-glutamine in monolayers at 37 °C in a humidified atmosphere of 5% CO_2_ [19], changing the medium twice a week. For all of the experiments, the Caco-2 cells at passage 45–60 were seeded in different plates and inserts, and were used 18–21 days post seeding, when completely differentiated.

### 2.3. MTT Viability Test

To ascertain any cytotoxic activity of the FA metabolites, the Caco-2 cell viability was assessed using an MTT assay [20]. The cells were seeded in 96-well plates (2.5 × 10^3^ cells/well in 100 μL) exposed to different concentrations (0.5, 1, 2.5, and 5 μM) of the compounds FA, FA glu, DHFA, DHFA glu, IFA, and IFA sulf, or to an equivalent volume of methanol for the controls, and were incubated for 48 h. At 24 h before treatments, the 10%-serum supplemented medium was discarded and replaced with a medium with 2.5% serum. After incubation, the medium was removed and 100 μL of the MTT solution (5 mg/mL of fresh serum-free medium) was added and left for 6 h at 37 °C. The medium was then discarded and 100 μL of DMSO was added in each well. Subsequently, the absorbance of each well was measured at 570 nm using a micro plate reader (Infinite 200, Tecan, Salzburg, Austria). Cell viability was expressed as percentage of control values.

### 2.4. Western Blot Analyses

To investigate the proteins by Western blot analyses, the samples were prepared as previously described [21]. In detail, Caco-2 cells were seeded in six-well plates (5 × 10^4^ cells/mL in 2 mL) in DMEM with 2.5% serum and 0.1 mM L-arginine. Once differentiated, the cells were pretreated with FA metabolites (1 μM for 30 min) or with an equivalent volume of methanol for the controls preceding LPS co-exposure (1 μg/mL) for 2–48 h, depending on the protein. Then, the medium was discarded, and the Caco-2 cells were lysed in 150 μL of a CellLytic M lysis buffer (Sigma Aldrich, Milan, Italy) combined with Pierce™ protease and phosphatase inhibitor mini tablets (Thermo Fisher Scientific, Waltham, MA, USA) for protein extraction. The lysates were centrifuged (12,500× *g*, 7 min, 4 °C), and then the supernatants were collected and used to calculate the total protein content using the Bradford assay [22] or were stored at −20 °C to be used for Western blot experiments. Then, 20 (for β-actin, IĸBα, Akt, and MAPKs detection) or 50 μg (for iNOS and Nrf2 detection) of reduced and denatured proteins were separated on 10% or 4–12% polyacrylamide gels, and were subsequently transferred into nitrocellulose membranes. Then, the membranes were blocked with 25 mL of TTBS (tris-buffered saline with Tween 20, composed of 100 mM NaCl, 0.1% Tween 20, and Tris/HCl 20 mM, pH 7.5) in 4% dry milk for 30 min at room temperature. Primary mono- and poly-clonal antibodies anti-ERK1/2 (#9102 CST), anti-phospho ERK1/2 (#4370 CST), anti-Nrf2 (#12721 CST), anti-p38, anti-phospho p38 (#9211 CST), anti-IĸBα (#4814 CST), anti-phospho IĸBα (#2859 CST), and β-actin (#4967 CST), and anti-iNOS (#13120 CST; Cell Signaling Technology, Inc., Danvers, MA, USA) in TTBS 1% dry milk (1:200–1:1000 depending on the manufacturing recommendation) were added to the membranes and left overnight on a three-dimensional rocking table at 4 °C. Subsequently, the membranes were washed twice with TTBS before incubation with rabbit secondary anti-mouse IgG (A9044, Sigma Aldrich, Milan, Italy) or with mouse anti-rabbit (sc-2357, Santa Cruz Biotechnology, Dallas, TX, USA), both conjugated to horseradish peroxidase (1:2000 dilution), in TTBS in 1% milk for 45 min at room temperature, and then washed twice with TTBS and once with TBS (100 mM NaCl and Tris/HCl 20 mM, pH 7.5). The bands were displayed by using the ChemiDoc™ XRS + System (Bio-Rad Laboratories, Inc., Hercules, CA, USA). The molecular weights of the protein bands were deduced from a comparison with pre-stained molecular weight markers that were run in parallel with the cell samples (range 14–180 kDa, GenScript, Piscataway, NJ, USA). Protein bands were dosed using Quantity One software (BioRad Laboratories).

### 2.5. Gene Expression by qRT-PCR

The total RNA was isolated from Caco-2 cells with QIAzol Lysis Reagent (Qiagen, Milano, Italy), according to the manufacturer’s instructions, and were quantified using NanoDrop ND1000 (Thermo Fisher Scientific, Monza, Italy). RNA was reverse-transcribed with a High Capacity cDNA Reverse Transcription Kit (Thermo Fisher Scientific) and the gene expression analysis was determined by quantitative real-time polymerase chain reaction (qRT-PCR) using a 7300 Real Time PCR system (Applied Biosystems) and specific TaqMan gene assays (Thermo Fisher Scientific) for Nrf2 (Hs00975961_g1) and NOS2 (iNOS) (Hs01075529_m1). Each sample was run in triplicate and human ACTB (Thermo Fisher Scientific) was used as the endogenous normalizer. The relative quantity was estimated using the ΔΔCt method, following the manufacturer’s instructions (Applied Biosystems).

### 2.6. Measure of NO and cGMP

To evaluate the NO and cGMP release, Caco-2 cells were seeded in six-well plates (5 × 10^4^ cells/mL in 2 mL) in phenol red-free DMEM with 2.5% serum and L-arginine 0.1 mM. Once differentiated, the cells were pretreated with FA metabolites (1 μM for 30 min) or with an equivalent volume of methanol for the controls preceding LPS co-exposure (1 μg/mL). After incubation, the medium was collected from each well and NO was measured as nitrite accumulation, determined by mixing 100 μL of the same medium with an equal volume of Griess’ reagent, and incubating for 20 min at room temperature. Then, the absorbance of each sample was detected at 540 nm, and the nitrite levels were calculated with a sodium nitrite standard curve (0.1 to 10 μM) [23]. The results were reported as μM of nitrites released by the cells. After removing the medium from the wells, the cell monolayers were treated with 300 μL of HCl 0.1 μM, and after 15 min they were scraped and centrifuged in small Eppendorf tubes to collect the supernatants for the cGMP analysis using the Cyclic GMP EIA Kit 96 well (Vinci-Biochem, Vinci, Italy).

### 2.7. Statistical Analyses

One-way analysis of variance (ANOVA) followed by Bonferroni’s test was performed to highlight the significant differences between groups (*p* < 0.05) using GraphPad Prism 5 software (GraphPad software, San Diego, CA, USA).

## 3. Results

### 3.1. Cell Viability

**Figure 1:**
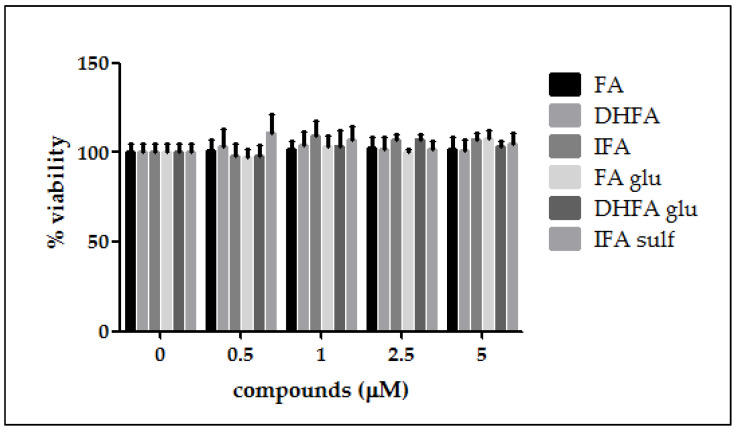
Percentage of cell viability compared with the control (0 μM, 100% viable cells) of Caco-2 cells incubated for 48 h with different concentrations of ferulic acid (FA), dihydroferulic acid (DHFA), isoferulic acid (IFA), ferulic acid glucuronide (FA glu), dihydroferulic acid glucuronide (DHFA glu), and isoferulic acid sulfate (IFA sulf; 0.5–5 μM). Each column represents the mean ± SD of the independent experiments (*n* = 12).

As a preliminary investigation before evaluating the FA metabolites’ efficacy against LPS deleterious effects in intestinal Caco-2 cells, we assessed the cell viability after treatment with different concentrations of the tested compounds. As reported in Figure 1, cell viability was not affected in the presence of all of the FA metabolites at all the tested concentrations (0.5–5 μM, 48 h).

### 3.2. Inhibition of iNOS Expression

**Figure 2:**
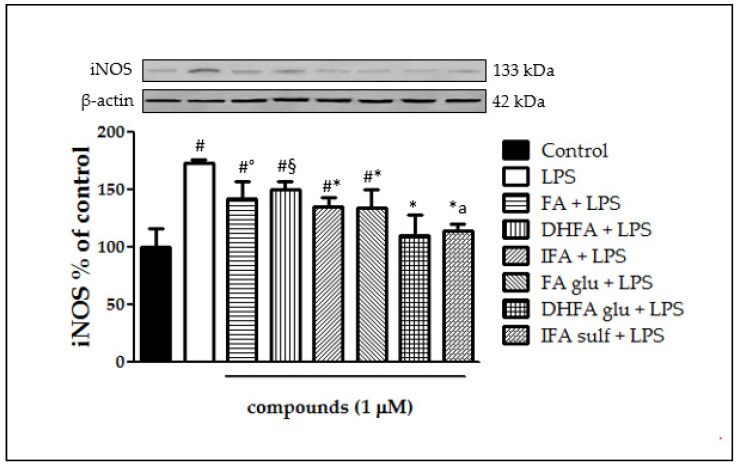
Level of iNOS dosed by Western blot and reported as percentage of the control, in Caco-2 cells treated with lipopolysaccharide (LPS) (1 μg/mL) for 48 h and pretreated for 30 min with FA, DHFA, IFA, FA glu, DHFA glu, and IFA sulf (1 μM) preceding LPS co-exposure. Control and LPS groups were pretreated with an equivalent volume of methanol. Each column represents the mean ± SD of the independent experiments (*n* = 6). Significant differences among groups are described using different superscript symbols; * = significant vs. LPS (*p* < 0.001); # = significant vs. control (*p* < 0.001); ° = significant vs. LPS, DHFA glu + LPS and IFA sulf + LPS (*p* < 0.01); § = significant vs. LPS (*p* < 0.05), DHFA glu + LPS and IFA sulf (*p* < 0.001); a = significant vs. FA glu + LPS and IFA + LPS (*p* < 0.05). A representative WB picture of the experiment is shown. β-actin detection was used as a loading control for each sample.

**Figure 3:**
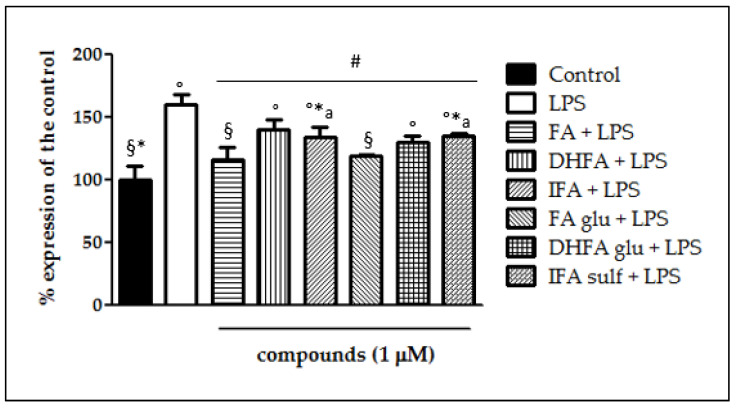
Expression of iNOS dosed by qRT-PCR and reported as a percentage of the control, in Caco-2 cells treated with LPS (1 μg/mL) for 48 h and pretreated for 30 min with with FA, DHFA, IFA, FA glu, DHFA glu, and IFA sulf (1 μM) prior to LPS co-exposure. Control and LPS groups were pretreated with an equivalent volume of methanol. Each column represents the mean ± SD of independent experiments (*n* = 6). Significant differences among groups are described using different superscript symbols; # = significant vs. LPS (*p* < 0.001); ° = significant vs. control (*p* < 0.001); * = significant vs. FA glu + LPS (*p* < 0.05); § = significant vs. DHFA + LPS (*p* < 0.001); a = significant vs. FA (*p* < 0.01).

The expression of iNOS plays a key role in maintaining the inflammatory state at the intestinal level. Figure 2 shows that a significant raise in iNOS expression occurred in LPS-treated cells compared with the control. In Caco-2 cells pretreated with the different isoforms and metabolites of FA, LPS-induced iNOS expression was significantly inhibited, albeit with differences in effectiveness between the tested compounds. Indeed, the phase I/II metabolites DHFA glu and IFA sulf not only exerted a significant effect (*p* < 0.001) in counteracting iNOS expression with respect to the LPS alone, but were also able to lead the enzyme levels to those of the untreated cells. Similar results were observed concerning the expression measured by qRT-PCR (Figure 3), where all of the compounds were able to significantly limit iNOS expression, sometimes leading them to control levels, as in the case of FA and its glucuronide.

### 3.3. Inhibition of NO Release

**Figure 4:**
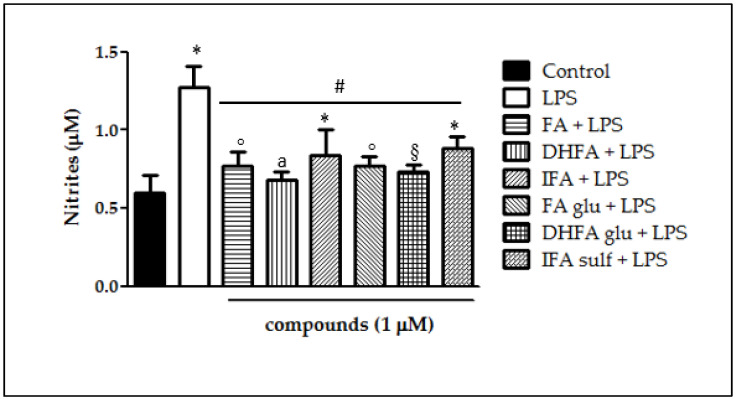
NO release (expressed as μM of nitrites) in Caco-2 cells treated with LPS (1 μg/mL) for 48 h and pretreated for 30 min with FA, DHFA, IFA, FA glu, DHFA glu, and IFA sulf (1 μM) preceding LPS co-exposure. Control and LPS groups were pretreated with an equivalent volume of methanol. Each column represents the mean ± SD of independent experiments (*n* = 16). Significant differences among groups are described using different superscript symbols; * = significant vs. control (*p* < 0.01); ° = significant vs. control (*p* < 0.05); # = significant vs. LPS (*p* < 0.001); § = significant vs. IFA sulf + LPS (*p* < 0.05); a = significant vs. IFA sulf + LPS (*p* < 0.001).

Following the increased expression of iNOS, we measured its main product NO as a common parameter of intestinal inflammation, together with its effector cGMP. NO release in Caco-2 cells after 48 h LPS exposure with and without pretreatment with the phenolic compounds (1 μM, 30 min preceding LPS co-exposure) was quantified as the nitrite content in the culture medium. As expected, LPS induced NO release, which doubled the NO levels produced by untreated cells (nitrites > 1 µM; Figure 4). All of the compounds were proven to significantly counteract LPS-induced NO production (nitrites < 1 µM). Interestingly, DHFA and its glucuronidated metabolite were the most successful at limiting the production of NO, for which the level was similar to that of the control cells.

### 3.4. Detection of cGMP Release

**Figure 5:**
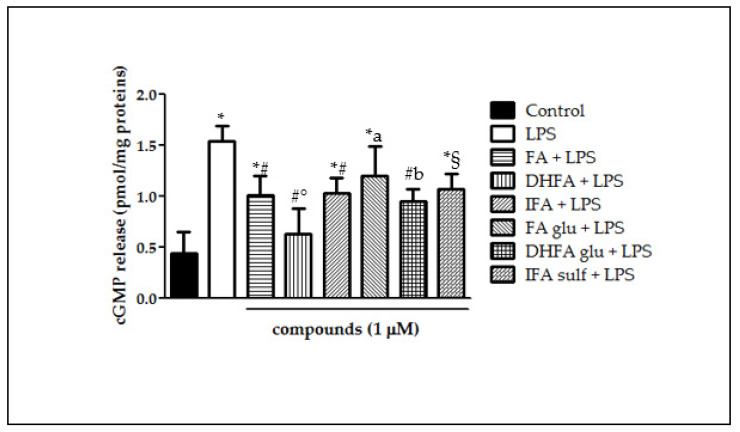
cGMP release (pmol/mg proteins) in Caco-2 cells incubated for 48 h with LPS (1 µg/mL) after pretreatment with FA, DHFA, IFA, FA glu, DHFA glu, and IFA sulf (1 μM) (*n* = 9). Control and LPS groups were pretreated with an equivalent volume of methanol. Significant differences among groups are described using different superscript symbols; * = significant vs. control (*p* < 0.001); # = significant vs. LPS (*p* < 0.001); ° = significant vs. IFA + LPS (*p* < 0.05); § = significant vs. LPS and DHFA + LPS (*p* < 0.01); a = significant vs. DHFA + LPS (*p* < 0.001); b = significant vs. control (*p* < 0.01).

LPS incubation in Caco-2 cells elicited NO production and consequently improved cGMP release by the guanylate cyclase, as shown in Figure 5. cGMP, measured as pmol/mg proteins, was in fact three-fold higher in LPS-treated cells in comparison with the untreated ones. Pretreatments with ferulates significantly reduced cGMP release, however showing differences in effectiveness. cGMP levels were shown to have lesser amounts particularly in those cells treated with DHFA and its glucuronide, reflecting what was observed for NO production. Differently, FA glu was able to limit cGMP release, but not to a statistically significant extent.

### 3.5. Prevention of IĸBα Decay

**Figure 6:**
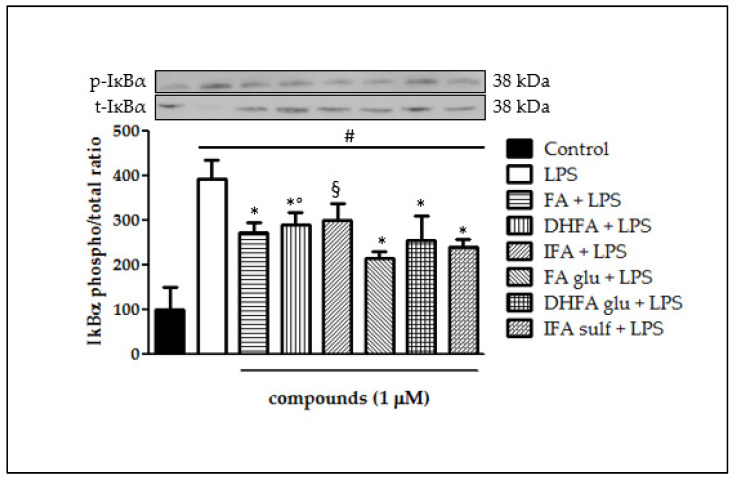
Degradation of IĸBα reported as percentage of the control phospho IĸBα/total IĸBα ratio in Caco-2 cells treated with LPS (1 μg/mL) for 48 h and pretreated for 30 min with FA, DHFA, IFA, FA glu, DHFA glu, and IFA sulf (1 μM) preceding LPS co-exposure. Control and LPS groups were pretreated with an equivalent volume of methanol. Each column represents the mean ± SD of independent experiments (*n* = 6). Significant differences among groups are described using different superscript symbols; * = significant vs. LPS (*p* < 0.001); # = significant vs. control (*p* < 0.001); ° = significant vs. FA glu + LPS (*p* < 0.05); § = significant vs. LPS and FA glu + LPS (*p* < 0.01). Representative WB picture of the experiment is shown.

IĸBα phosphorylation and its consequent degradation are main events that follow the activation of upstream kinases and leads to NF-ĸB translocation into the cell nucleus. This process is critical in the coupling of extracellular stimuli to the transcriptional activation of specific target genes. Figure 6 reports IĸBα degradation which took place in Caco-2 cells after incubation with LPS for 48 h. The ratio between total IĸBα and its phosphorylated form was assessed through Western blot analysis. IĸBα phosphorylation was notable in cells treated with LPS alone (four-fold increase of phospho/total IĸBα ratio) with respect to control cells, while pretreatment with all FA metabolites significantly prevented IĸBα phosphorylation and thus its decay.

### 3.6. Modulation of Akt

**Figure 7:**
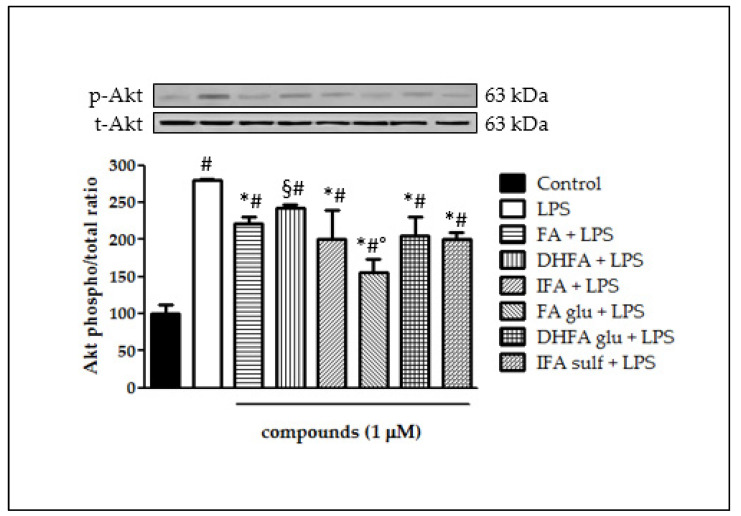
Activation of Akt reported as percentage of the control phospho Akt/total Akt ratio in Caco-2 cells treated with LPS (1 μg/mL) for 2 h and pretreated for 30 min with FA, DHFA, IFA, FA glu, DHFA glu, and IFA sulf (1 μM) preceding LPS co-exposure. Control and LPS groups were pretreated with an equivalent volume of methanol. Each column represents the mean ± SD of independent experiments (*n* = 6). Significant differences among groups are described using different superscript symbols; # = significant vs. control (*p* < 0.001); * = significant vs. LPS (*p* < 0.001); § = significant vs. LPS (*p* < 0.05) and vs. FA glu + LPS (*p* < 0.001); ° = significant vs. FA + LPS, IFA + LPS, DHFA glu + LPS and IFA sulf + LPS (*p* < 0.01). Representative WB picture of the experiment is shown.

The ability of FA metabolites to regulate the intracellular signaling pathways involved in NF-ĸB activation was evaluated, starting from the kinase Akt. As shown in Figure 7, incubation with LPS resulted in a significant rise in the Akt kinase phosphorylation state after 2 h of incubation (three-fold increase). All of the FA isomers and metabolites negatively modulated Akt phosphorylation, as the phospho Akt/total Akt ratio was reduced in all samples incubated with FA, IFA, DHFA glu, FA glu, IFA sulf (*p* < 0.001), and DHFA (*p* < 0.05). Among these compounds, the most active was found to be FA glu, which kept the phospho/total Akt ratio at around 1 (two-fold increase).

### 3.7. Reduction of MAPK p38 and ERK1/2 Activation

**Figure 8:**
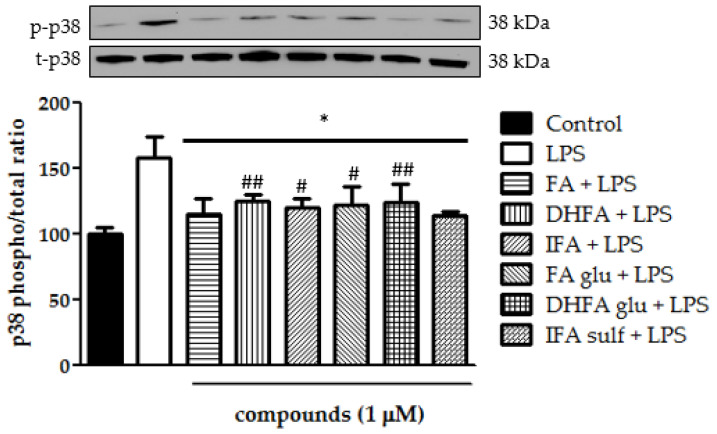
Modulation of MAPK p38 reported as a percentage of the control phospho p38/total p38 ratio in Caco-2 cells treated with LPS (1 μg/mL) for 2 h and pretreated for 30 min with FA, DHFA, IFA, FA glu, DHFA glu, and IFA sulf (1 μM) preceding LPS co-exposure. Control and LPS groups were pretreated with an equivalent volume of methanol. Each column represents the mean ± SD of the independent experiments (*n* = 6). Significant differences among groups are described using different superscript symbols; * = significant vs. LPS (*p* < 0.001); # = significant vs. control (*p* < 0.05); ## = significant vs. control (*p* < 0.01). Representative WB picture of the experiment is shown.

**Figure 9:**
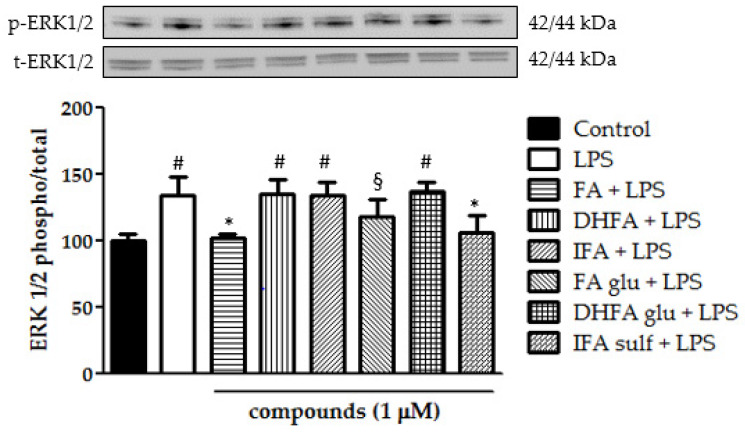
Modulation of MAPK ERK1/2 reported as a percentage of the control phospho ERK1/2/total ERK1/2 ratio in Caco-2 cells treated with LPS (1 μg/mL) for 2 h and pretreated for 30 min with FA, DHFA, IFA, FA glu, DHFA glu, and IFA sulf (1 μM) preceding LPS co-exposure. Control and LPS groups were pretreated with an equivalent volume of methanol. Each column represents the mean ± SD of independent experiments (*n* = 6). Significant differences among groups are described using different superscript symbols; * = significant vs. LPS (*p* < 0.001); # = significant vs. control, FA + LPS and IFA sulf + LPS (*p* < 0.001); § = significant vs. FA + LPS (*p* < 0.05). Representative WB picture of the experiment is shown.

Still, in the context of the kinases that are involved in the activation of NF-ĸB, we analyzed ERK1/2 and p38 phosphorylation after treatment with LPS and ferulates for 2 h. Concerning p38, it was seen that all of the tested compounds were significantly able to limit LPS-induced phosphorylation of this MAPK (Figure 8). Going more into detail, FA and IFA sulf reduced the levels of p38 phosphorylation, even leading them back to those of untreated cells. The incubation with LPS also resulted in a significant boost of ERK1/2 phosphorylation (Figure 9). In this case, the compounds that were found to be the most significantly active against the phosphorylation of p38 (namely FA and IFA sulf), were the only ones capable of limiting, even partially, the phosphorylation of ERK1/2 (*p* < 0.001). The remaining FA metabolites, on the other hand, showed no significant effect.

### 3.8. Expression of Nrf-2

**Figure 10:**
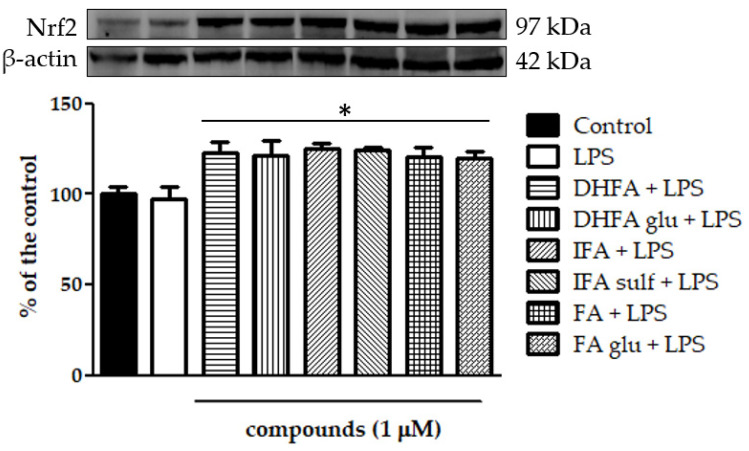
Level of Nrf2 dosed by Western blot and reported as a percentage of the control in Caco-2 cells treated with LPS (1 μg/mL) for 48 h and pretreated for 30 min with FA, DHFA, IFA, FA glu, DHFA glu, and IFA sulf (1 μM) preceding LPS co-exposure. Control and LPS groups were pretreated with an equivalent volume of methanol. Each column represents the mean ± SD of independent experiments (*n* = 6). Significant differences among groups are described using different superscript symbols; * = significant vs. control (*p* < 0.001) and LPS (*p* < 0.001). Representative WB picture of the experiment is shown. β-actin detection was used as a loading control for each sample.

**Figure 11:**
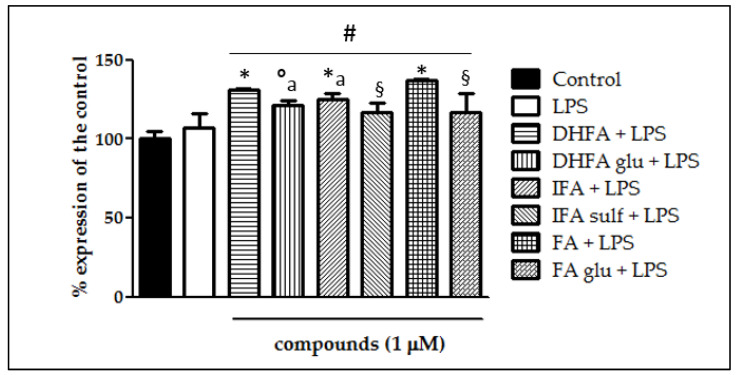
Expression of Nrf2 dosed by qRT-PCR and reported as a percentage of the control in Caco-2 cells treated with LPS (1 μg/mL) for 6 h and pretreated for 30 min with FA, DHFA, IFA, FA glu, DHFA glu, and IFA sulf (1 μM) preceding LPS co-exposure. Control and LPS groups were pretreated with an equivalent volume of methanol. Each column represents the mean ± SD of independent experiments (*n* = 6). Significant differences among groups are described using different superscript symbols; **#** = significant vs. control (*p* < 0.001); * = significant vs. LPS (*p*< 0.001); ° = significant vs. LPS (*p* < 0.01); § = significant vs. FA + LPS (*p* < 0.001) and vs. DHFA + LPS (*p* < 0.01); a = significant vs. IFA + LPS and DHFA glu + LPS (*p* < 0.01).

The expression of Nfr2 contributes to the anti-inflammatory process by arranging the recruitment of inflammatory cells, inhibiting NF-ĸB activation by preventing the degradation of IĸB-α, and improving antioxidant defenses. In this study, we treated Caco-2 cells with LPS for 6 h and 48 h to evaluate Nrf2 expression and level, measured by qRT-PCR and Western blot, in the presence/absence of FA and its metabolites. Cells treated only with LPS did not show any significant increase and/or decrease of the Nrf2 expression with respect to the untreated ones (Figure 10 and Figure 11), while the co-treatment with FA and its derivatives was instead significantly effective (*p* < 0.001). Some slight differences among the compounds were seen in the gene expression analysis (Figure 11), where FA and its derivative DHFA showed the greatest increase in Nrf2 expression.

## 4. Discussion

Defects of the intestinal epithelial barrier function are a feature of IBD, which present, as a characteristic trait, an aberrant activation of different signaling pathways leading to the expression of specific cytokines and enzymes, all involved in the onset and progression of the inflammatory processes [24]. It has been demonstrated that some compounds of a natural origin with anti-inflammatory and antioxidant activities are able to limit the inflammatory response by acting on several pathways, especially those upstream of the NF-ĸB expression. Conversely, little is known about phase I and II metabolites that are normally formed in vivo, which are usually present at higher concentrations in the circulation and can therefore be responsible for the biological activities generally attributed to their parent compounds [25]. The present study thus aimed to explore the ability of dietary FA and its metabolites to act, through different mechanisms, as anti-inflammatory agents in intestinal cells through the modulation of NF-ĸB-linked pathways involved in the overexpression of proinflammatory mediators.

Compounds were tested at 1 μM to mimic the concentrations that can be found in the gut lumen after the dietary intake of FA [26]. Caco-2 cell monolayers were used as a model of human intestinal epithelium for measuring the expression of proinflammatory enzymes such as iNOS and signaling related to the NF-ĸB activity and Nrf-2 [27,28,29]. These cells were incubated with the pro-inflammatory agent LPS at a pathological concentration of 1 μg/mL [30], which is suitable for inducing a proinflammatory response without severely interfering with the cell viability [21,27,28]. LPS was chosen to stimulate Caco-2 because it is abundant in the intestinal lumen, being the major cell wall component of Gram-negative bacteria, and it correlates with the pathogenesis of IBD when reaching elevated concentrations [31,32]. Having ascertained that treatments were non-toxic for Caco-2 cells at the tested concentrations of LPS and ferulates, the upregulated release of NO and cGMP, associated with the expression of the enzyme iNOS, was then measured in Caco-2 cells monolayers. It was seen that LPS induced a significant increase of iNOS gene expression and protein concentrations (*p* < 0.001) with respect to the untreated cells, and the co-treatment with all the ferulates inhibited such a rise. In the cells challenged with LPS and IFA sulf or DHFA glu, the values were led back to those of the controls. NO release by iNOS and the consequent cGMP release were then measured in the cell supernatant after 48 h of LPS exposure. NO production, measured as the nitrite concentration, was two-fold higher in those cells treated only with LPS, with respect to the controls, while the cGMP release was even three-fold higher. NO production was considerably reduced in cells pretreated with the tested compounds and the same results were observed in the case of cGMP release. The abnormal cGMP production after iNOS overexpression at the intestinal level caused by LPS is known to contribute to enhancing intestinal permeability and diarrhea in ulcerative colitis [9], thus, keeping lower levels thanks to the presence of these phenolic compounds may be an important tool to maintain gut homeostasis [33]. In addition, the anti-inflammatory effects of FA derivatives, albeit measured with different parameters, have also been recently studied in macrophages [34,35], which are also involved in the maintenance of the inflammatory processes in the gut [36].

Once we observed the inhibition of inflammatory response by ferulates in intestinal cells, we aimed to determine which upstream molecular signaling pathways might be involved.

The activity of NF-ĸB was then evaluated in relation to the degradation of its inhibitor IĸB. Indeed, the disappearance of IĸB (especially the subunit IĸBα) allows NF-ĸB to translocate into the nucleus to induce the expression of several NF-ĸB-dependent genes, such as iNOS and COX-2 [37]. As reported elsewhere with similar experimental protocols [27,28,38], a potent NF-ĸB activator such as LPS induced the degradation of IĸBα after 48 h of incubation through phosphorylation, which was four-fold higher than in the control cells, whereas all the FA derivatives partially limited this process. It is interesting to note that the phase II metabolite IFA sulf was even more effective than its precursor IFA, and the result can be explained by evaluating the concomitance of effects occurring upstream of the phosphorylation of IĸBα. This inhibitor usually gets phosphorylated by its kinase IKK, which, in turn, could be activated in the early stages (30 min–2 h) by other kinases [39,40]. Among these kinases, PI3K/Akt-mediated p65 phosphorylation plays an important role in Gram-negative enteric bacteria-induced NF-ĸB activation. Moreover, the activation of the Toll-like receptor (TLR) 4 by LPS has also led to the activation of all MAPK pathways, which in turn activates the phosphorylating cascade that drives the activity of NF-ĸB [37]. In this regard, the functional modifications of Akt and of the MAPK p38 and ERK1/2 proteins have been assessed in Caco-2 cells after 2 h of incubation with LPS. As already verified by our research group [21,28], LPS significantly stimulated activation through the phosphorylation of Akt (+170%), ERK1/2, and p38 (+30–50%). Pretreatment with the metabolites of FA, albeit with some differences in activity, was able to limit the LPS-induced phosphorylation of all three kinases. In particular, FA and IFA sulf resulted in the most successful in decrease of p38 and ERK1/2 phosphorylation, while FA glu showed the best effect in hampering the Akt pathway. Globally, these results suggest that the effectiveness of these molecules against the hyperactivation of NF-ĸB could be at least partly linked to their interaction with these upstream signals. To confirm this hypothesis, however, further studies that directly link these two mechanisms are needed.

Another pathway that was recently proposed for the deactivation of NF-ĸB is that of Nrf2, which is known to be able to prevent the degradation of IĸBα and, at the same time, to increase antioxidant defenses to neutralize oxidative stress and turn off the inflammatory processes [11]. Likewise, the activation of NF-ĸB in turn regulates the expression of Nrf2, thus justifying the theory of a crosstalk between the two signaling pathways [36]. In the present investigation, the Nrf2 levels were dosed as the protein concentration by Western blot and as the gene expression by qRT-PCR. In the cells challenged with LPS, neither an increase nor a decrease of the Nrf2 expression was observed, confirming the results recently reported by Wu et al. [29]. Co-treatment with FA and its metabolites instead significantly improved both the Nrf2 gene expression and protein concentration: some of the compounds, such as the free form FA and its derivative DHFA, were found to be more effective than the other metabolites at increasing the gene expression, while all the compounds were equally effective at enhancing the protein concentration. In RAW264.7 cell macrophages, FA was instead shown to reduce the Nrf2 expression in the context of NF-ĸB modulation [41]. This difference in the outcome may be related to the cell type and the redox state of the cells. Likely, an FA concentration of 100 µM used by Lampiasi et al. [41] in their interesting investigation markedly suppressed oxidative stress, thus inhibiting the expression of Nrf2, which is strongly linked to the levels of oxidative stress in cells. Conversely, in this study, LPS did not significantly increase oxidative stress, and the compounds at a 100-times lower concentration (1 µM) did not affect the redox state compared with the untreated cells (data not shown). Therefore, it is possible to propose a reasonable mechanism of action that is partially or totally unrelated to the antioxidant capacity of these compounds and especially of the FA phase II metabolites, which may lose the scavenging properties after their conjugation with glucuronide/sulfate/methylate moieties [18,25]. In any case, to better evaluate the effects of these compounds in vivo, it is necessary to take into account their actual bioaccessibility, which is influenced by the metabolism at different levels (endothelial, intestinal, hepatic, etc.), the transport mechanisms, and the possible deconjugation that may occur once they reach the target tissues [42]. Some of these aspects cannot be evaluated in vitro as shown here, and must therefore be further investigated in vivo. Research on animal models and clinical trials will be necessary to determine if the promising activities verified in this work are clinically reflected in the improvements of the chronic intestinal inflammatory state, especially in patients with IBD.

## 5. Conclusions

This study pointed out for the first time the positive effects of the principal phase I/II FA metabolites against intestinal inflammation acting on different signaling routes. Specifically, the inhibition of the IĸB degradation by FA metabolites was reflected in the decrease of the LPS-induced iNOS expression and, in turn, of the NO and cGMP levels. These compounds were proven to be able to deactivate the MAPK and Akt kinase signaling pathways, and to increase the expression of Nrf2, all key factors that may have downregulated the NF-ĸB activity. The results confirm that the metabolic conversion of dietary FA into its metabolites does not undermine the bioactivity of the free form, but on the contrary originates compounds equally capable of preserving intestinal integrity against pro-inflammatory agents, thus hampering or limiting the progression of intestinal inflammation and related diseases.

## Data Availability

The data presented in this study are available on request from the corresponding author.
